# Radiosynthesis and Preclinical Evaluation of a Novel ^11^C‑Labeled Pyrazolopyrimidine Derivative for Positron Emission Tomography Imaging of Phosphodiesterase 2A

**DOI:** 10.1021/acsmedchemlett.5c00649

**Published:** 2025-12-12

**Authors:** Yinlong Li, Wakana Mori, Zhendong Song, Tomoteru Yamasaki, Taoqian Zhao, Jiahui Chen, Yiding Zhang, Xin Zhou, Lin Xie, Tomomi Kokufuta, Kuan Hu, Qilong Hu, Masayuki Fujinaga, Xiaoyan Li, Katsushi Kumata, Chongjiao Li, Zhenkun Sun, Yabiao Gao, Danielle E. Hoyle, Jimmy S. Patel, Hongjie Yuan, Ming-Rong Zhang, Steven H. Liang

**Affiliations:** ⊥ Department of Radiology and Imaging Sciences, 1371 Emory University, 1364 Clifton Road, Atlanta, Georgia 30322, United States; ‡ Department of Advanced Nuclear Medicine Sciences, Institute for Quantum Medical Sciences, National Institutes for Quantum Science and Technology, Chiba 263-8555, Japan; § Department of Pharmacology and Chemical Biology, Emory University School of Medicine, Atlanta, Georgia 30322, United States; ∥ Department of Radiation Oncology, Winship Cancer Institute of Emory University, Atlanta, Georgia 30322, United States

**Keywords:** Phosphodiesterase 2A, Positron emission tomography, Radioligand, Carbon-11, Brain imaging

## Abstract

Phosphodiesterase 2A (PDE2A) plays a vital role in regulating cyclic nucleotide signaling by hydrolyzing cAMP and cGMP in the central nervous system (CNS). This enzymatic activity is essential for neuronal function, and PDE2A has emerged as a molecular target for neuroimaging in neuropsychiatric disorders and neurodegenerative diseases. In this study, we evaluated the novel ^11^C-labeled positron emission tomography (PET) radioligand [^11^C]**1** derived from a pyrazolopyrimidine-based PDE2A inhibitor. The radiosynthesis of [^11^C]**1** was accomplished via [^11^C]­methyl iodide-mediated methylation of precursor **9** under mild conditions, yielding [^11^C]**1** with high purity (99%) and high molar activity (154 ± 66 GBq/μmol). *In vitro* autoradiography demonstrated high radiotracer accumulation in regions with abundant PDE2A expression, including the striatum and substantia nigra. However, dynamic PET imaging in rats showed a relatively uniform distribution throughout the brain and no significant blocking effects. Further optimization in medicinal chemistry is necessary to improve the *in vivo* performance of the pyrazolopyrimidine-based PDE2A tracer scaffold.

## Graphical abstract

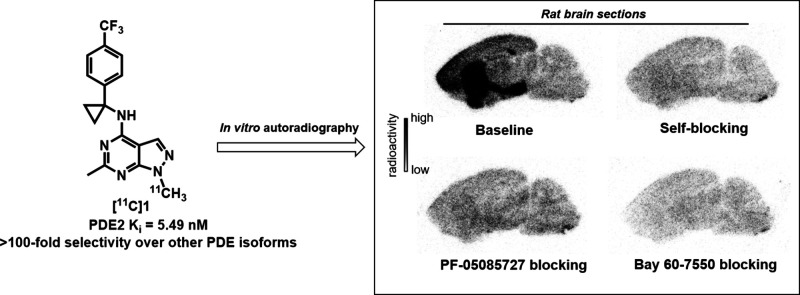

Cyclic adenosine 3′,5′-monophosphate (cAMP) and cyclic guanosine 3′,5′-monophosphate (cGMP) function as essential second messengers that regulate various intracellular signaling pathways, including those regulating calcium homeostasis and neurotransmission.^1,2^ Phosphodiesterases (PDEs) forms a superfamily of enzymes responsible for degrading cAMP and/or cGMP by hydrolyzing their cyclic phosphate bonds, thereby modulating numerous physiological processes.^3,4^ The PDE superfamily comprises 11 subtypes and can be categorized by substrate selectivity: PDE4, PDE7, and PDE8 preferentially hydrolyze cAMP, PDE5, PDE6, and PDE9 are cGMP-selective, while PDE1, PDE2, PDE3, PDE10, and PDE11 act on both substrates.^5–7^ These dual-substrate PDEs are considered to play a critical role in coordinating the crosstalk between cAMP and cGMP signaling networks.^8,9^ Among them, PDE2 (particularly the PDE2A isoform) is of particular interest due to its high distribution within the central nervous system (CNS).^10–13^ PDE2A is widely expressed in the striatum, cortex, hippocampus, and substantia nigra, where it modulates neuronal excitability, synaptic plasticity, and cognitive function.^14,15^ Dysregulation of PDE2A activity has been linked to various neuropsychiatric and neurodegenerative conditions, such as Alzheimer’s disease (AD),^16–18^ major depressive disorder,^19^ and schizophrenia.^20^ In recent years, several selective PDE2A inhibitors such as BAY 60-7550,^21^ PF-05085727,^22^ and TAK-915 ^23^ have been developed and demonstrated promising results in preclinical studies. However, none of these compounds have advanced to clinical approval, highlighting the need for continued development of PDE2A-targeted therapeutics.

While positron emission tomography (PET) holds promise as a molecular imaging modality for visualizing the distribution and function of PDE2A *in vivo*, its application remains largely investigational, with ongoing efforts focused on the development and validation of suitable radiotracers. (Figure 1).^24–26^ [^18^F]**B-23** represents the first potent PDE2A (IC_50_ = 1 nM) PET radioligand, but its modest selectivity against PDE10A (IC_50_ = 11 nM) and substantial brain-penetrant radiometabolites limited its utility in translational imaging studies.^11,27^ [^18^F]**PF-05270430** (IC_50_ = 0.5 nM), developed by Pfizer Inc., exhibited high target-specificity in the striatum of nonhuman primates (NHPs) and was subsequently advanced to clinical trials.^28^ However, quantitative analysis using the cerebellum as a reference region resulted in a relatively low estimated binding potential.^29^ Based on a triazine scaffold, Schröder *et al.* explored [^18^F]**TA3** (IC_50_ = 11.4 nM), [^18^F]**TA4** (IC_50_ = 7.3 nM), and [^18^F]**TA5** (IC_50_ = 3.0 nM) for PET imaging of PDE2A.^30,31^ However, these tracers were not suitable for neuroimaging applications as high levels of nonspecific binding and the accumulation of brain-penetrant radiometabolites were observed. Similarly, triazine-based radioligand [^18^F]**BIT1** was developed, but its nonspecific binding observed *in vivo* limited its further evaluation.^32^ Recently, a triazolopyridopyrazine-based radioligand [^18^F]**11** was reported.^33^ However, its *in vivo* evaluation showed minimal regional-specific uptake, with comparable accumulation in PDE2A-enriched and reference regions (e.g., caudate putamen vs cerebellum). Despite extensive efforts, a clinically validated PET radioligand for PDE2A imaging remains scarce.^34^ Herein, we report the development and preclinical evaluation of a new pyrazolopyrimidine-based analog [^11^C]**1** as a potential PDE2A PET ligand (Figure 2).

**Figure 1:**
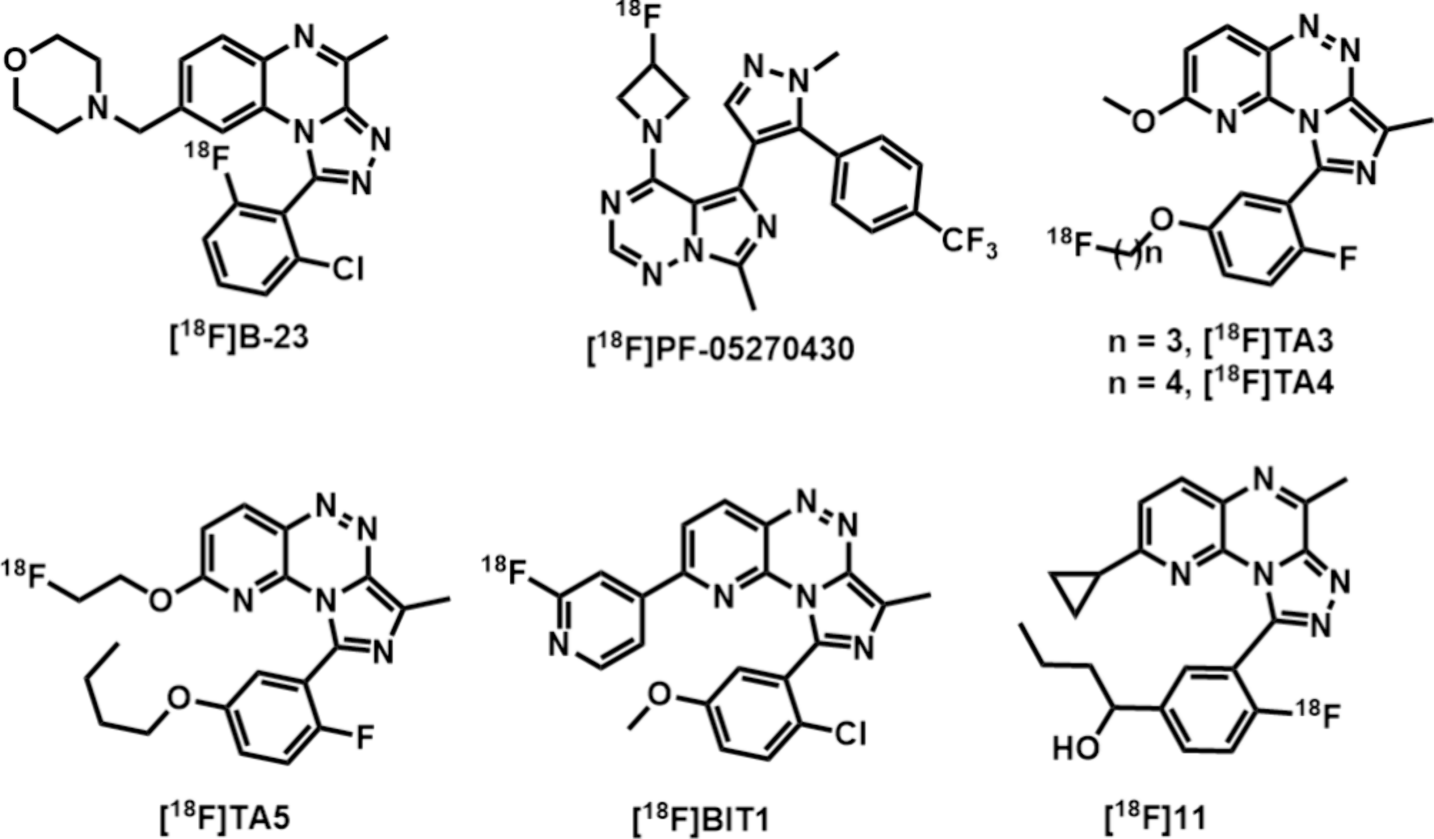
Potential PDE2A PET ligands.

**Figure 2:**
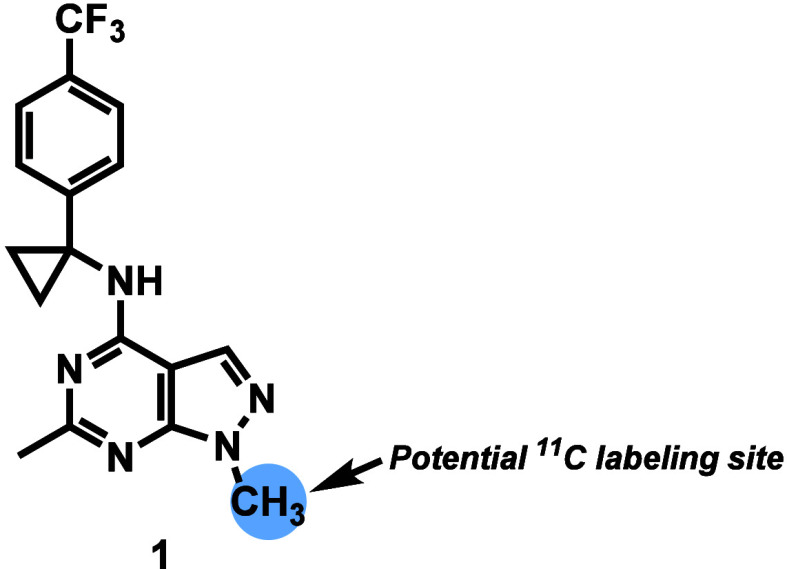
Chemical structure of the novel PDE2A ligand **1** (this work).

## Materials and Methods

### General

Unless specified otherwise, all commercial reagents were purchased from commercial sources and used as received without additional purification. Compound **1** and its corresponding precursor **9** were synthesized according to the published method.^35^ NMR spectra were obtained using a Bruker AVANCE NEO 400 MHz spectrometer (400 MHz for proton (^1^H) and 101 MHz for carbon (^13^C) spectra). Chemical shifts (δ) are reported in parts per million (ppm), referenced to tetramethylsilane (TMS) via the residual solvent signal. High-performance liquid chromatography–mass spectrometry (HPLC-MS) was performed using a Shimadzu LCMS-2010EV single four-stage rod mass spectrometer ESI electric spray ion source or Agilent single four-stage rod 6120B ESI electric spray ion source. Analytical HPLC was carried out on an Agilent 1100 system equipped with a G1315A detector. High-resolution mass spectrometry (HRMS) was carried out on an AB Sciex TripleTOF 5600+ LC/MS using electrospray ionization (ESI^+^). All animal procedures were approved by the ethical guidelines of Emory University and National Institutes for Quantum Science and Technology.

### Chemistry

#### 1,6-Dimethyl-1*H*-pyrazolo­[3,4-*d*]­pyrimidin-4-ol (**3**)

To a solution of compound **2** (1.00 g, 7.14 mmol) in dioxane (10 mL) was added dropwise DIEA (1.01 g, 7.85 mmol) and acetyl chloride (616 mg, 7.85 mmol) at 0 °C. The mixture was stirred at 0 °C for 5 min, then an additional 10 min after removal from the ice water bath. The mixture was stirred at 70 °C for 48 h under N_2_. The mixture was then heated to 110 °C and stirred for 4 h. A mixture of Na_2_CO_3_ (1.51 g, 14.3 mmol) in dioxane (5.00 mL) was added slowly, and the resulting suspension was stirred at 110 °C for 1 h under N_2_. The reaction mixture was diluted with EtOAc (20 mL) and washed with H_2_O (20 mL × 3). The combined organic layers were washed with brine (50.0 mL × 2), dried over Na_2_SO_4_, filtered and concentrated under reduced pressure to give compound **3** (585 mg, 58% yield, 99.4% purity) as a white solid. ^1^H NMR (400 MHz, MeOD): δ 7.98 (s, 1H), 3.94 (s, 3H), 2.46 (s, 3H). LCMS: *m*/*z* = 164.9 (M + H)^+^.

#### 4-Chloro-1,6-dimethyl-1*H*-pyrazolo­[3,4-*d*]­pyrimidine (**4**)

To a solution of compound **3** (585 mg, 3.56 mmol) in POCl_3_ (16.5 g, 107 mmol, 10 mL) at 0 °C. Then the mixture was stirred at 150 °C for 10 h under N_2_. The reaction mixture was quenched by the addition of H_2_O (20 mL) and then extracted with NaHCO_3_ (25 mL) and CH_2_Cl_2_ (50 mL × 2). The combined organic layers were washed with brine (100 mL × 2), dried over Na_2_SO_4_, filtered and concentrated under reduced pressure to give compound **4** (1.30 g, crude) as a yellow oil. ^1^H NMR (400 MHz, MeOD) δ 8.61 (s, 1H), 4.14 (s, 3H), 2.81 (s, 3H). LCMS: *m*/*z* = 182.9 (M + H)^+^.

#### 1,6-Dimethyl-*N*-(1-(4-(trifluoromethyl)­phenyl)­cyclopropyl)-1*H*-pyrazolo­[3,4-*d*]­pyrimidin-4-amine (**1**)

To a solution of compound **4** (100 mg, 548 μmol), compound **5** (110 mg, 548 μmol) in NMP (3 mL) was added DIEA (354 mg, 2.74 mmol). Then the mixture was stirred at 100 °C for 18 h under N_2_. The reaction mixture was diluted with EtOAc (20 mL) and washed with H_2_O (20 mL × 3). The combined organic layers were washed with brine (50 mL × 2), dried over Na_2_SO_4_, filtered and concentrated under reduced pressure to give a residue. The residue was purified by prep-HPLC (column: Welch Xtimate C_18_ 40 × 200 mm, 7 μm; mobile phase: [water (NH_3_·H_2_O + NH_4_HCO_3_)/ACN]; gradient: 22%–62% B over 25 min) to give compound **1** (71.0 mg, 37% yield for 2 steps, 99.9% purity) as a white solid. ^1^H NMR (400 MHz, CDCl_3_) δ 7.53 (d, *J* = 8.4 Hz, 2H), 7.41 (s, 1H), 7.28 (s, 2H), 6.34 (s, 1H), 3.94 (s, 3H), 2.60 (s, 3H), 1.59 (s, 4H). ^13^C NMR (101 MHz, CDCl_3_) δ 165.04, 158.38, 155.16, 146.23, 133.43, 125.89, 128.91 (q, *J* = 65 Hz), 125.37, 123.76, 122.67, 119.97, 97.96, 36.51, 33.77, 25.88, 22.91. LCMS: *m*/*z* = 348.1 (M + H)^+^. HRMS: *m*/*z* [M + H]^+^ calcd for C_17_H_17_F_3_N_5_^+^, 348.1431; found, 348.1439.

#### *tert*-Butyl 4-Chloro-6-methyl-1*H*-pyrazolo­[3,4-*d*]­pyrimidine-1-carboxylate (**7**)

To a mixture of compound **6** (670 mg, 3.97 mmol) and (Boc)_2_O (1.04 g, 4.77 mmol) in THF (10 mL) was added DMAP (97.1 mg, 794 μmol), TEA (804 mg, 7.95 mmol) at 25 °C under N_2_. The mixture was stirred at 25 °C for 5 h under N_2_. The reaction mixture was quenched by the addition of H_2_O (80 mL) at 25 °C, and then extracted with EtOAc (160 mL). The combined organic layers were washed with brine (80 mL), dried over Na_2_SO_4_, filtered, and concentrated under reduced pressure to give compound **7** (965 mg) as a white solid. ^1^H NMR (400 MHz, CDCl_3_) δ (ppm) 8.23 (s, 1H), 2.90 (s, 3H), 1.74 (s, 9H). LCMS: *m*/*z* = 268.9 (M + H)^+^.

#### *tert*-Butyl 6-Methyl-4-((1-(4-(trifluoromethyl)­phenyl)­cyclopropyl)­amino)-1*H*-pyrazolo­[3,4-*d*]­pyrimidine-1-carboxylate (**8**)

To a mixture of compound **7** (500 mg, 1.86 mmol) and compound **5** (299 mg, 1.49 mmol) in NMP (10 mL) was added DIEA (1.20 g, 9.30 mmol) at 25 °C under N_2_. The mixture was stirred at 100 °C for 5 h. The reaction mixture was quenched by the addition of H_2_O (80 mL) at 25 °C, and then extracted with EtOAc (160 mL). The combined organic layers were washed with brine (80 mL), dried over Na_2_SO_4_, filtered, and concentrated under reduced pressure to give a residue. The residue was purified by flash silica gel chromatography (ISCO; 40 g SepaFlash Silica Flash Column, eluent of 0–10% CH_2_Cl_2_/MeOH at 18 mL/min) to give compound **8** (420 mg) as a yellow oil and was directly used for the next step. LCMS: *m*/*z* = 434.0 (M + H)^+^.

#### 6-Methyl-*N*-(1-(4-(trifluoromethyl)­phenyl)­cyclopropyl)-1*H*-pyrazolo­[3,4-*d*]­pyrimidin-4-amine (**9**)

To a mixture of compound **8** (420 mg, 969 μmol) in TFA (5.00 mL) at 25 °C under N_2_. The mixture was stirred at 25 °C for 12 h. The reaction mixture was concentrated under reduced pressure to give a residue. The residue was purified by prep-HPLC (column: F-Prepulite XP tC 18 40 × 200 mm × 7 μm; mobile phase: [water (TFA)/ACN]; gradient: 2–42% B over 20.5 min) to give compound **9** (34.6 mg, 11% yield for 3 steps) as a white solid. ^1^H NMR (400 MHz, CDCl_3_) δ (ppm) 8.14–8.29 (m, 1H), 7.56 (br d, *J* = 8.0 Hz, 3H), 7.30 (br d, *J* = 8.4 Hz, 2H), 2.63 (s, 3H), 1.64 (br s, 4H). ^13^C NMR (101 MHz, CDCl_3_) 167.33, 163.59, 158.28, 155.87, 145.53, 135.69, 129.14, 126.02, 125.31, 123.74, 122.61, 97.64, 36.80, 34.21, 22.33. LCMS: *m*/*z* = 334.2 (M + H)^+^. HRMS: *m*/*z* [M + H]^+^ calcd for C_16_H_15_F_3_N_5_^+^, 334.1274; found, 334.1281.

### Radiochemistry

The irradiations were performed on a CYPRIS HM-18 (Sumitomo Heavy Industries, Tokyo, Japan) (18 MeV protons). [^11^C]­CO_2_ was produced via the ^14^N­(p,α)^11^C nuclear reaction using a high-purity nitrogen gas target containing 0.01% O_2_. The radiosynthesis of the [^11^C]**1** was carried out using an automated synthesis system developed in-house.^36^ In brief, [^11^C]­CO_2_ was bubbled into 0.4 *M* LiAlH_4_ in anhydrous THF (300 μL). After evaporation of the THF, the remaining complex was treated with 57% hydroiodic acid (300 μL) to give [^11^C]­CH_3_I, which was distilled at 180 °C and transferred by N_2_ gas into a solution of **9** (1.0 mg), TBAH (tetrabutylammonium hydroxide, 1 *M* in MeOH, 6.0 μL), and DMF (250 μL) at −15 to −20 °C. After radioactivity reached a plateau, this reaction mixture was heated at 30 °C for 5 min. Then, the HPLC buffer (CH_3_CN/H_2_O = 55/45, 0.1% Et_3_N, 5.0 mL) was added and then injected into HPLC for purification (column: YMC Triart C18 column, 10 × 250 mm; buffer: CH_3_CN/H_2_O = 55/45, 0.1% Et_3_N; flow rate: 5.0 mL/min). The total synthesis time was 31 min from the end of bombardment (EOB). [^11^C]**1** was obtained in 28 ± 13% radiochemical yield (*n* = 5) with excellent radiochemical purity (99%) and molar activity (154 ± 66 GBq/μmol, *n* = 5).

### *In Vitro* Autoradiography

Briefly, brain sections (20 μm) from male Sprague-Dawley (SD) rats were incubated with [^11^C]**1** (33.1 MBq/L) in a buffer containing 50 mM Tris-HCl (pH 7.4), 2 mM MgCl_2_, 1.2 mM CaCl_2_ at room temperature for 30 min (*n* = 4). For blocking studies, 1 μM of either cold compound **1**, PF-05085727, or BAY 60-7550 was added to the incubation buffer. After incubation, sections were washed three times (2 min each) with prechilled buffer and briefly rinsed in cold distilled water (10 s). The slices were then air-dried and placed on imaging screens for 60 min before being scanned using a BAS5000 system (Fujifilm).

### PET Imaging

Dynamic PET imaging was performed using an Inveon PET scanner (Siemens). Approximately 32 MBq of [^11^C]**1** (*n* = 2 in each group) was administered intravenously via a tail vein catheter of the SD rat. For the blocking study, unlabeled **1** (1 mg/kg) was administrated 5 min before the injection of [^11^C]**1**. Scans were acquired in 3D mode over 60 min under 1.5% isoflurane anesthesia, with body temperature maintained using a T-pump system. Images were reconstructed using a Hanning filter with a Nyquist cutoff of 0.5 cycles/pixel, with the following framing: 4 × 1 min, 8 × 2 min, and 8 × 5 min. Data analysis was performed using PMOD version 3.4.

### *Ex Vivo* Whole-Body Distribution

In brief, [^11^C]**1** (4.6 MBq) was administered intravenously via the tail vein to ddY mice (male, *n* = 3). At the time points of 1, 5, 15, 30, and 60 min, the mice were euthanized by cervical dislocation, and selected tissues were harvested and weighed. Decay-corrected radioactivity in each organ was measured using a Wizard automatic gamma counter (PerkinElmer, USA).

### Radiometabolite Analysis

Male SD rats were intravenously injected with [^11^C]**1** (63 MBq, 0.1 mL) and sacrificed by decapitation at 10-and 30 min postinjection (*n* = 2 at each time point). Brain and plasma samples were rapidly collected and processed following previously reported procedures.^37^ An aliquot of the supernatant (0.1–0.5 mL) obtained from the plasma or brain was injected into the HPLC system equipped with YMC-Triant C18 column (5 μm, 4.6 × 250 mm) and a radiodetector at a flow rate of CH_3_CN/H_2_O (70/30 v/v, containing 0.1% Et_3_N) at a flow rate of 1.0 mL/min. The percentage of intact [^11^C]**1** was calculated as follows: % = (peak area for [^11^C]**1**/total peak area) × 100. The HPLC recovery was 86.2% ± 5.8 for plasma and 92.3% ± 2.0 for brain samples.

## Results and Discussion

### Chemistry and Physiochemical and Pharmacological Properties

As shown in Scheme 1, the intermolecular reaction of amine **2** with acetyl chloride afforded pyrimidine **3** in 58% yield. Chlorination of the hydroxy group in compound **3** using POCl_3_ produced chloride **4**, which then underwent S_N_Ar reaction with amine **5** to yield compound **1** in an overall yield of 37% over two steps.

**Scheme 1:**
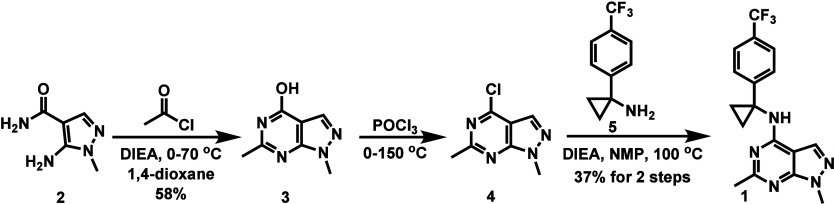
Synthesis of Compound **1**

The pharmacology and ADME parameters for compound **1** are summarized in Table 1. Compound **1** shows a PDE2A binding affinity (*K*_i_ = 5.49 nM) with >100-fold selectivity over other PDE subtypes.^35^ Compound **1** has a molecular weight of 347.35 (<500) and a calculated LogP (cLogP) of 3.53. The experimentally measured logD of 3.88 is favorable for blood–brain barrier (BBB) penetration.^38^ The topological polar surface area (tPSA) of 52.35 (<90) and one hydrogen bond donor (HBD) indicate good drug-like properties. Moreover, compliance with Lipinski’s rule of five, with zero violations, suggests good oral bioavailability. In addition, the logBB value (−0.23) and CNS MPO score (5.1) further support BBB penetration, while the unbound brain fraction (*f*_u_ brain = 7.2%) demonstrates low probability of high nonspecific binding.^39,40^ All these properties indicate that compound **1** has favorable characteristics for *in vivo* evaluation.

**Table 1:**
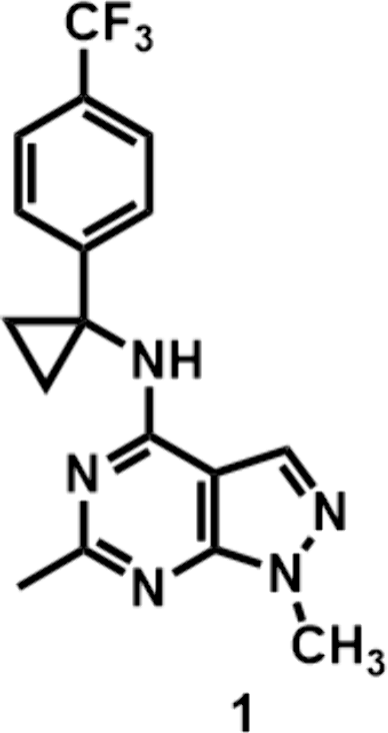
Pharmacology and ADME Parameters for Compound **1** ^a^

property	value
PDE2A *K*_i_ (nM)	5.49
MW^a^	347.35
clogP^a^	3.53
logD^b^	3.88
tPSA^a^	52.35
HBD^c^	1
Lipinski’s rule^c^	0
logBB^c^	–0.23
MPO score^c^	5.1
*f*_u_ brain (%)	7.2

a Calculated with ChemDraw 21.0 software.

b Measured by the “shake flask” method with LC-MS/MS analysis.

c Predicted with ACD/labs.

### Radiochemistry

The radiosynthesis of [^11^C]**1** commenced with the preparation of the desmethyl precursor **9**. As illustrated in Scheme 2, protection of the nitrogen in pyrimidine **6** with a Boc group afforded compound **7**. An S_N_Ar reaction of **7** with amine **5** yielded compound **8**, followed by Boc deprotection using TFA to give compound **9** in an overall yield of 11% over three steps. The radiosynthesis of [^11^C]**1** was achieved via a TBAH-promoted carbon-11 methylation of compound **9** at 30 °C for 5 min (Scheme 3). The resulting radiotracer [^11^C]**1** was produced in a radiochemical yield (RCY) of 28%, with a radiochemical purity of 99% and a molar activity of 154 ± 66 GBq/μmol (*n* = 5) at the end of synthesis (EOS). The identity of [^11^C]**1** was confirmed by HPLC coinjection with the corresponding nonradioactive reference compound (Figure S1).

**Scheme 2:**
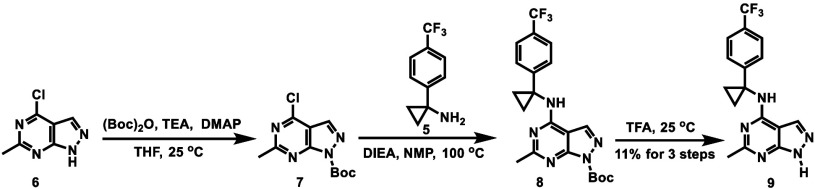
Synthesis of Precursor **9**

**Scheme 3:**
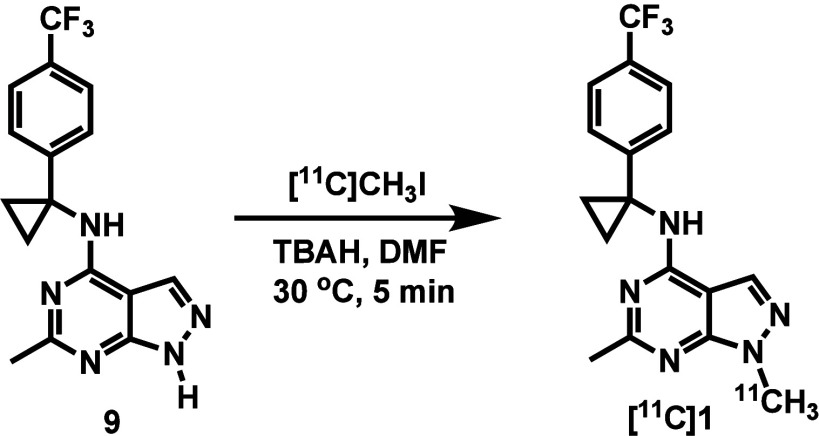
Radiosynthesis of [^11^C]**1**

### *In Vitro* Autoradiography Studies

To evaluate the binding specificity of tracer [^11^C]**1**, we first conducted *in vitro* autoradiography studies on SD rat brain sections (Figure 3). [^11^C]**1** exhibited a heterogeneous distribution pattern, with the high tracer accumulation in the cortex, striatum, hippocampus and substantia nigra. At the same time, lower radioactivity was observed in the cerebellum and pons. This distribution pattern is consistent with the documented regional expression of PDE2A in the rodent brain. Co-incubation with either unlabeled compound **1** (1 μM) or validated PDE2A inhibitors PF-05085727 (1 μM) and BAY 60-7550 (1 μM) significantly reduced the radiotracer signal in PDE2A-rich regions, leading to a more homogeneous distribution. In contrast, the cerebellum and pons showed minimal changes under blocking conditions. These findings demonstrate that [^11^C]**1** possesses high *in vitro* binding specificity for PDE2A, supporting its potential for *in vivo* PET imaging application.

**Figure 3:**
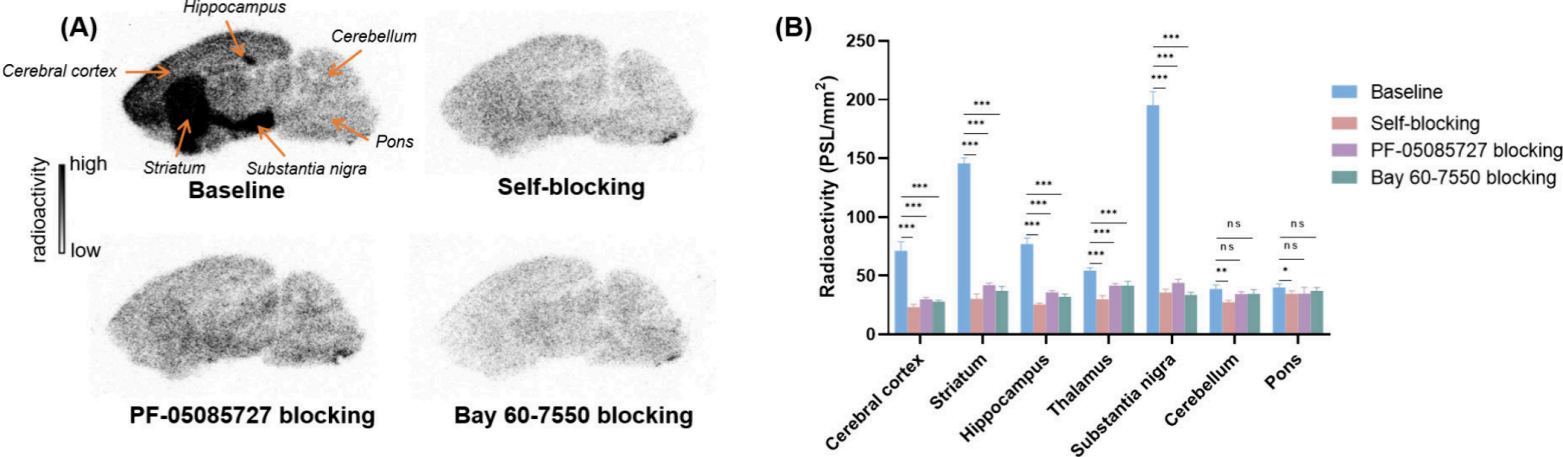
(A) Representative *in vitro* autoradiograms of [^11^C]**1** on SD rat sagittal brain sections under baseline and blocking conditions. (B) Quantification of autoradiography results for the regions of interest. Asterisks (*) indicate statistical significance: ns = not significant, *p ≤ 0.05, **p ≤ 0.01, ***p ≤ 0.001. Data are shown as mean ± SD.

### *In Vivo* PET Imaging Study

Given the promising *in vitro* binding of [^11^C]**1**, we performed dynamic *in vivo* PET imaging studies in SD rats (Figure 4). Under baseline conditions, summed PET images (3–20 min) revealed a homogeneous distribution across coronal, sagittal, and axial planes (Figure 4A, left). Time–activity curves (TACs) showed rapid brain uptake (SUV_max_ = 1.65 at 2 min postinjection) followed by fast clearance, with SUVs decreased below 0.5 by 20 min (Figure 4B,C). In self-blocking experiments, preadministration of unlabeled compound **1** (1 mg/kg) led to similar TAC patterns and regional brain distributions to baseline, indicating low *in vivo* binding affinity. This discrepancy between *in vitro* strong heterogeneous binding and *in vivo* weak homogeneous signals may be attributed to radiometabolic instability or high nonspecific binding caused by insufficient target affinity or high lipophilicity of [^11^C]**1**.

**Figure 4:**
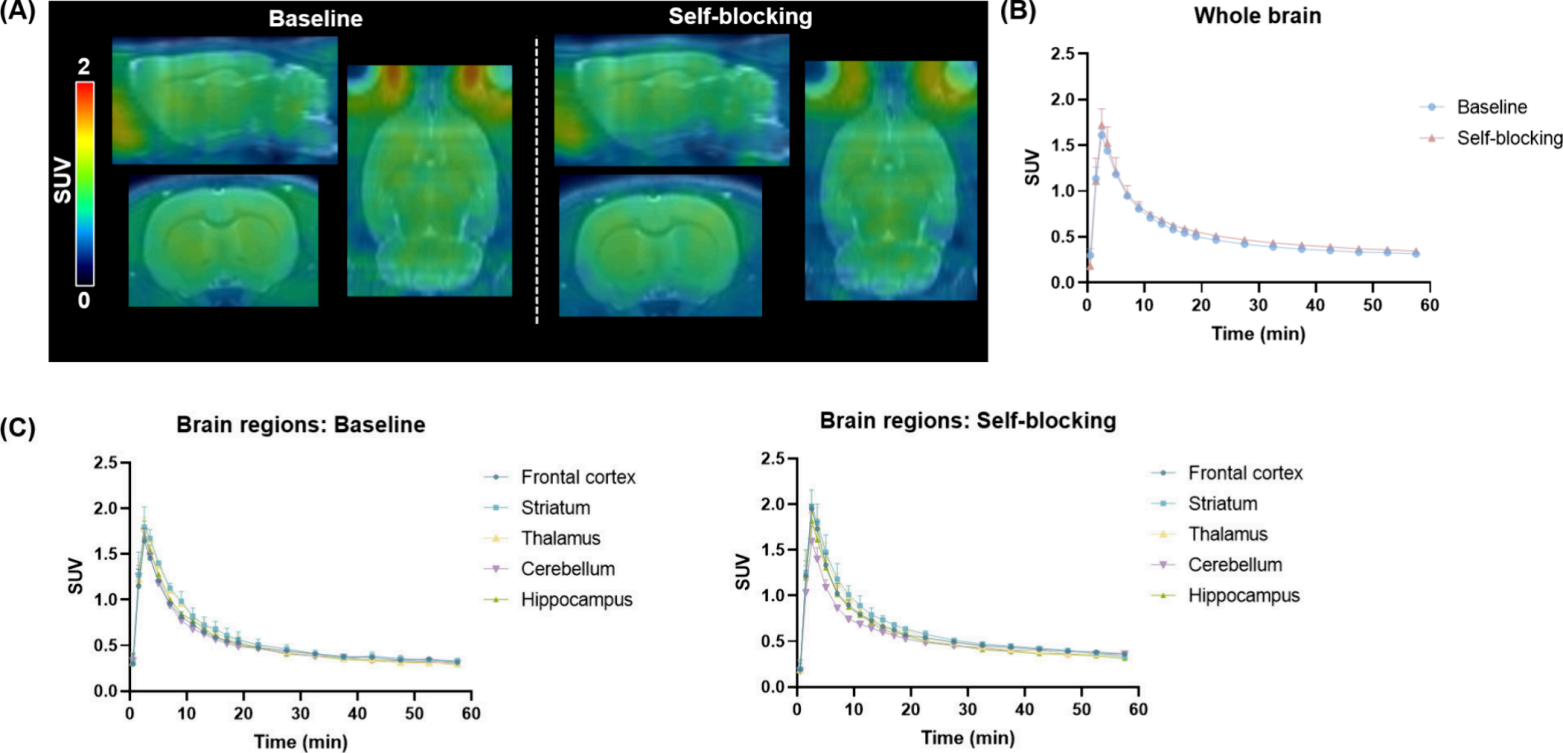
(A) Representative PET images (3–20 min) of [^11^C]**1** in rat brains under baseline and self-blocking conditions (1 mg/kg of cold compound **1**). (B) TACs of [^11^C]**1** in the whole brain. (C) Regional TACs of [^11^C]**1** in selected brain areas. Data are shown as mean ± SD.

### Whole-Body Biodistribution Study

*Ex vivo* biodistribution studies of [^11^C]**1** were performed in ddY mice at 1, 5, 15, 30, and 60 min postinjection (Figure 5). [^11^C]**1** demonstrated rapid clearance from the brain, consistent with PET imaging data. The tracer distributed quickly to peripheral organs, with high initial uptake in the liver, kidneys, adrenals, and small intestine, suggesting predominant hepatobiliary clearance. Notably, small intestine uptake markedly increased over time, reaching 11 ± 1.5% ID/g at 60 min, consistent with radiotracer excretion. Uptake in other tissues, including the heart, lung, and testis, was moderate and declined gradually.

**Figure 5:**
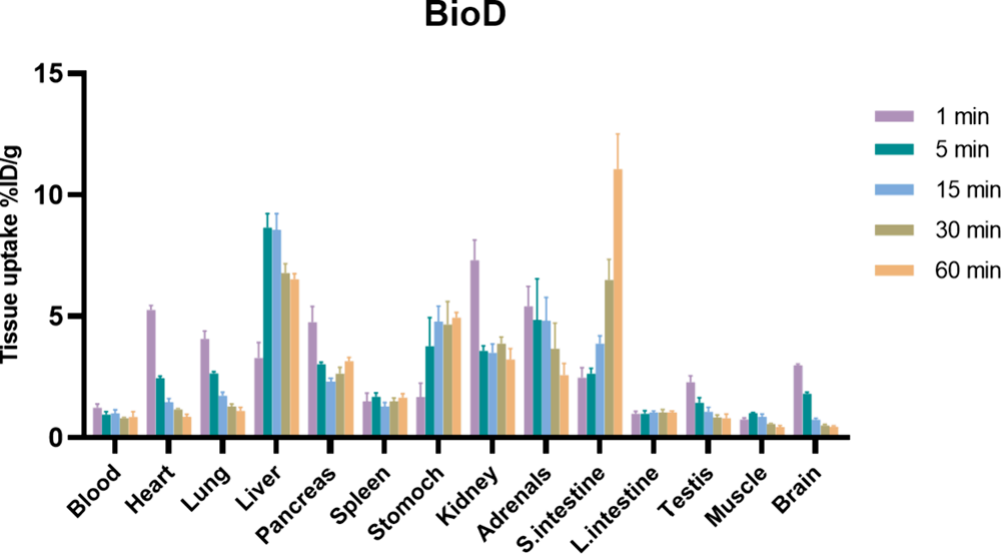
*Ex vivo* whole-body biodistribution (BioD) of [^11^C]**1** in ddY mice at 1, 5, 15, and 60 min postinjection. Data are shown as mean ± SD.

### Radiometabolite Analysis

*Ex vivo* radiometabolite analysis of [^11^C]**1** in SD rats revealed high metabolic stability in the brain, with 97% and 86% of the parent compound remaining at 10- and 30- min postinjection, respectively (Figure 6). In contrast, plasma metabolism of [^11^C]**1** was more rapid, with the percentage of unchanged [^11^C]**1** decreasing from 54% at 10 min to 34% at 30 min. These results indicate that [^11^C]**1** remains largely intact in the brain over the imaging period, despite moderate peripheral metabolism. The poor *in vivo* PET performance is unlikely attributed to metabolic instability to a large extent, but rather suggests that higher binding affinity or improved pharmacokinetic properties may be required.

**Figure 6:**
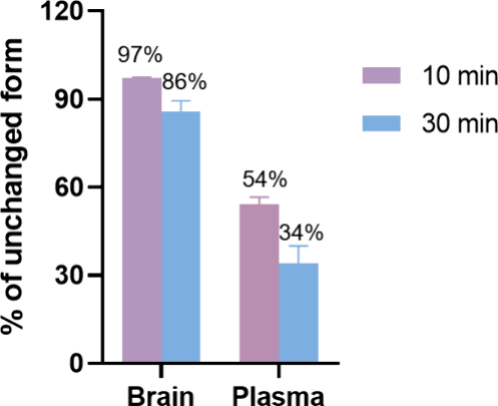
Radiometabolic analysis of [^11^C]**1** in SD rat brain and plasma at 10 and 30 min postinjection. Data are shown as mean ± SD.

## Conclusion

A ^11^C-labeled PET radioligand [^11^C]**1** based on a literature compound was developed as a potential tracer with high affinity and selectivity for imaging PDE2A and the ability to cross the blood–brain barrier. The radiosynthesis of [^11^C]**1** was achieved *via* N-[^11^C]­methylation, yielding the tracer in good radiochemical yield and high molar activity. *In vitro* autoradiography on rat brain sections showed specific accumulation in PDE2A-rich regions. However, dynamic *in vivo* PET imaging in rats revealed limited target-specific signal, indicating that [^11^C]**1** is not suitable for CNS imaging of PDE2A. These results highlighted the challenge of translating *in vitro* binding potential into robust *in vivo* imaging performance. Further SAR optimization to improve the binding affinity and overall pharmacokinetic properties of PDE2A-targeted tracers is currently in progress.

## Safety Statement

No unexpected or unusually high safety hazards were encountered.

## Supplementary Material



## Notes

NMR spectra of compounds **1**, **3**, **4**, **7**, and **9**; HPLC, MS, and HRMS analyses of compounds **1** and **9**; HPLC chromatographic analysis of [^11^C]**1** and HPLC coinjection analysis of [^11^C]**1** with the corresponding nonradioactive reference compound (Figure S1) (PDF)

## Abbreviations

PDE2A: phosphodiesterase 2A
CNS: central nervous system
PET: positron emission tomography
cAMP: cyclic adenosine 3′,5′-monophosphate
cGMP: cyclic guanosine 3′,5′-monophosphate
PDE: phosphodiesterase
AD: Alzheimer’s disease
NMR: nuclear magnetic resonance
ppm: parts per million
^1^H: proton
^13^C: carbon-13
TMS: tetramethylsilane
HPLC-MS: high-performance liquid chromatography–mass spectrometry
HRMS: high-resolution mass spectrometry
ESI^+^: positive-mode electrospray ionization
DIEA: *N*,*N*-diisopropylethylamine
EtOAc: ethyl acetate
N_2_: nitrogen
NMP: *N*-methyl-2-pyrrolidone
ACN: acetonitrile
THF: tetrahydrofuran, DMAP, 4-dimethylaminopyridine
TEA: triethylamine
TFA: trifluoroacetic acid
TBAH: tetrabutylammonium hydroxide
DMF: dimethylformamide
EOB: end-of-bombardment
SD rat: Sprague-Dawley rat
S_N_Ar: nucleophilic aromatic substitution reaction
ADME: absorption, distribution, metabolism, and excretion
BBB: blood–brain barrier
tPSA: topological polar surface area
HBD: hydrogen-bond donor
MPO: multiparameter optimization desirability
RCY: radiochemical yield
EOS: end of synthesis
TAC: time–activity curve
SUV: standardized uptake value
BioD: biodistribution
