# Supplementary material for: Radiosynthesis and Preclinical Evaluation of a Novel 11C‑Labeled Pyrazolopyrimidine Derivative for Positron Emission Tomography Imaging of Phosphodiesterase 2A
Source: ACS Med Chem Lett. 2025 Dec 12;17(1):218–25. doi: 10.1021/acsmedchemlett.5c00649 (PMC12794084; doi:10.1021/acsmedchemlett.5c00649)
Supplement: Supplementary file 1 [file ml5c00649_si_001.pdf]

Supporting Information

Radiosynthesis and Preclinical Evaluation of a Novel ^{11}C-labeled Pyrazolopyrimidine Derivatives for Positron Emission Tomography Imaging of Phosphodiesterase 2A

Yinlong Li,[1,†] Wakana Mori,[2,†] Zhendong Song,[1] Tomoteru Yamasaki,[2] Taoqian Zhao,[1] Jiahui Chen,[1] Yiding Zhang,[2] Xin Zhou,[1] Lin Xie,[2] Tomomi Kokufuta,[2] Kuan Hu,[2] Qilong Hu,[1] Masayuki Fujinaga,[2] Xiaoyan Li,[1] Katsushi Kumata,[2] Chongjiao Li,[1] Zhenkun Sun,[3] Yabiao Gao,[1] Danielle E. Hoyle,[3] Jimmy S. Patel,[1,4] Hongjie Yuan,[3] Ming-Rong Zhang,[2,*] Steven H. Liang[1,*]

[1]Department of Radiology and Imaging Sciences, Emory University, 1364 Clifton Road, Atlanta, Georgia 30322, United States

[2]Department of Advanced Nuclear Medicine Sciences, Institute for Quantum Medical Sciences, National Institutes for Quantum Science and Technology, Chiba 263-8555, Japan

[3]Department of Pharmacology and Chemical Biology, Emory University School of Medicine, Atlanta, Georgia, 30322, United States

[4]Department of Radiation Oncology, Winship Cancer Institute of Emory University, Atlanta, Georgia, 30322, United States

[†]These authors contributed equally

*Corresponding authors: M-R. Zhang: zhang.ming-rong@qst.go.jp. S.H. Liang: steven.liang@emory.edu

Content

1) NMR spectra of compounds **1**, **3**, **4**, **7**, and **9**
2) HPLC, MS and HRMS analyses of compounds **1**, and **9**
3) HPLC chromatographic analysis of [^{11}C]**1** and HPLC co-injection analysis of [^{11}C]**1** with the corresponding non-radioactive reference compound

^{1}H NMR spectrum of compound **3**

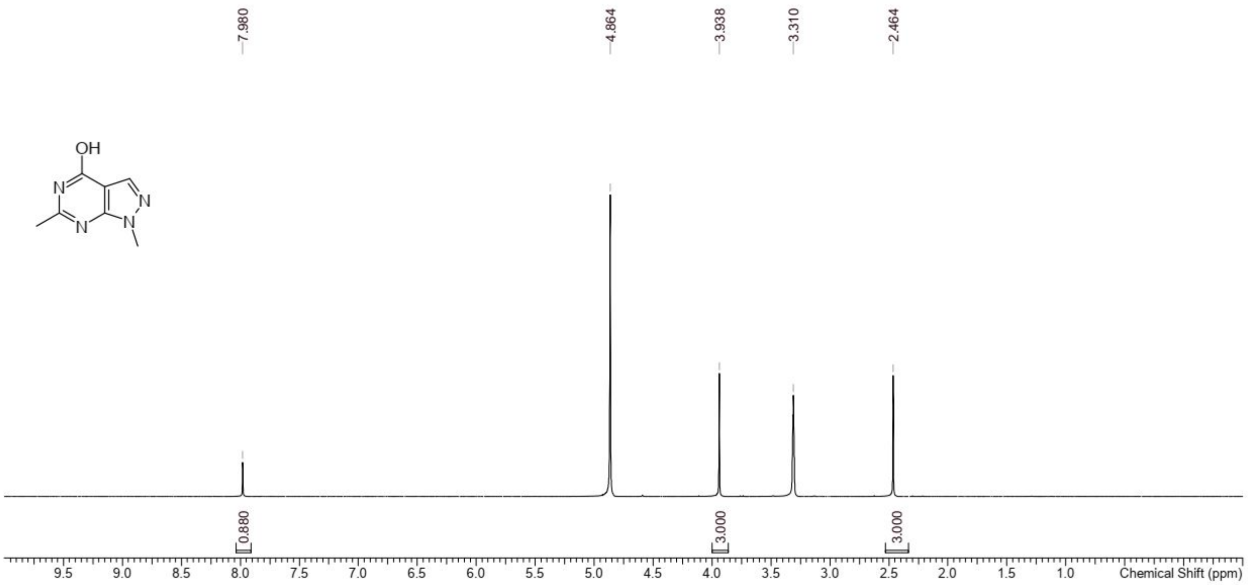

^{1}H NMR spectrum of compound **4**

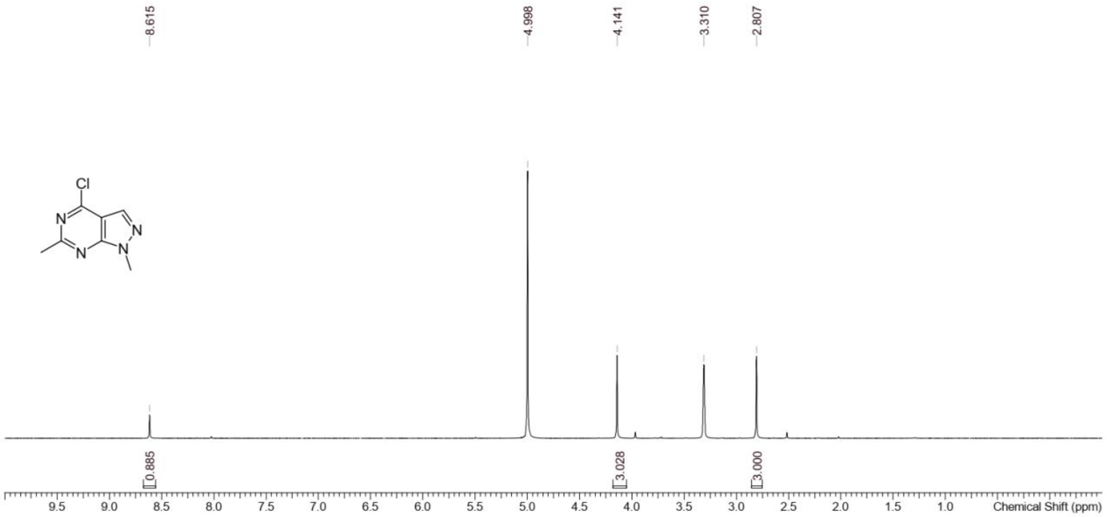

^{1}H NMR spectrum of compound **1**

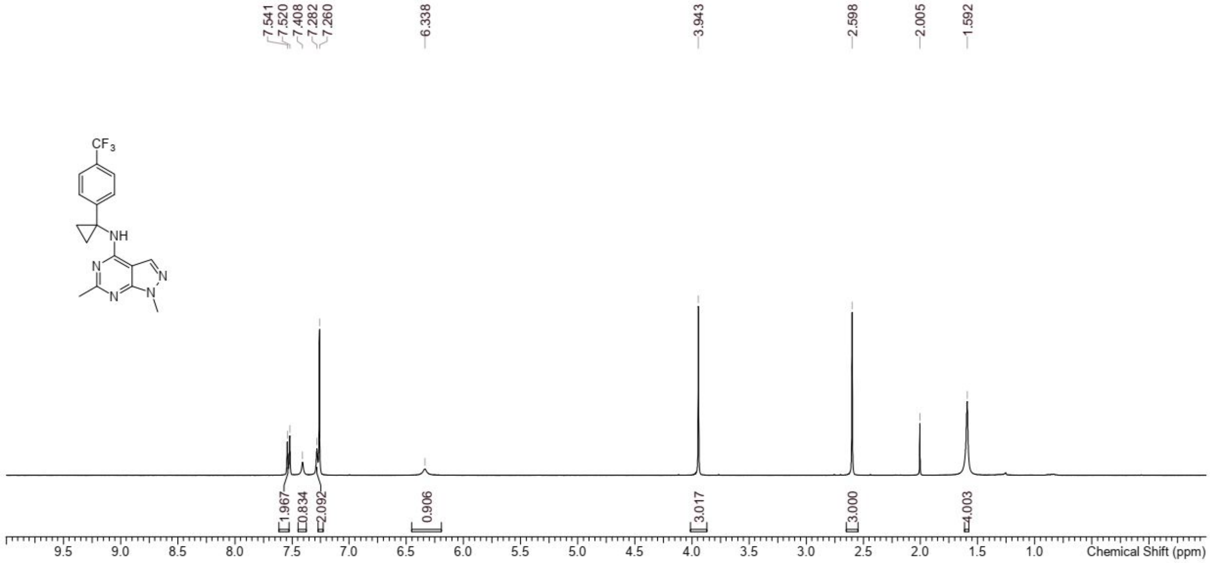

^{1}H NMR spectrum of compound **7**

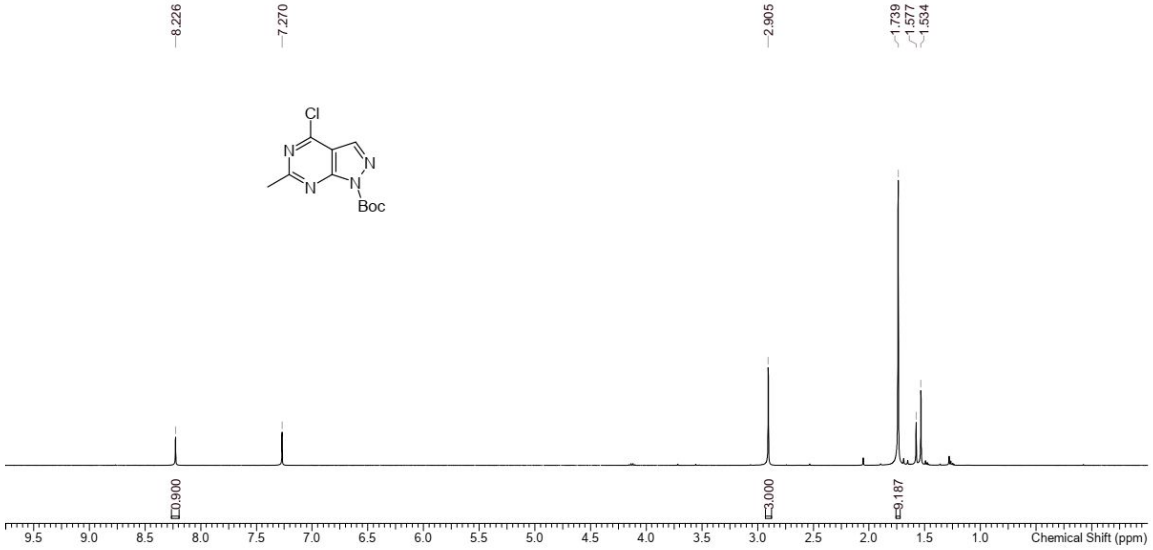

^{1}H NMR spectrum of compound **9**

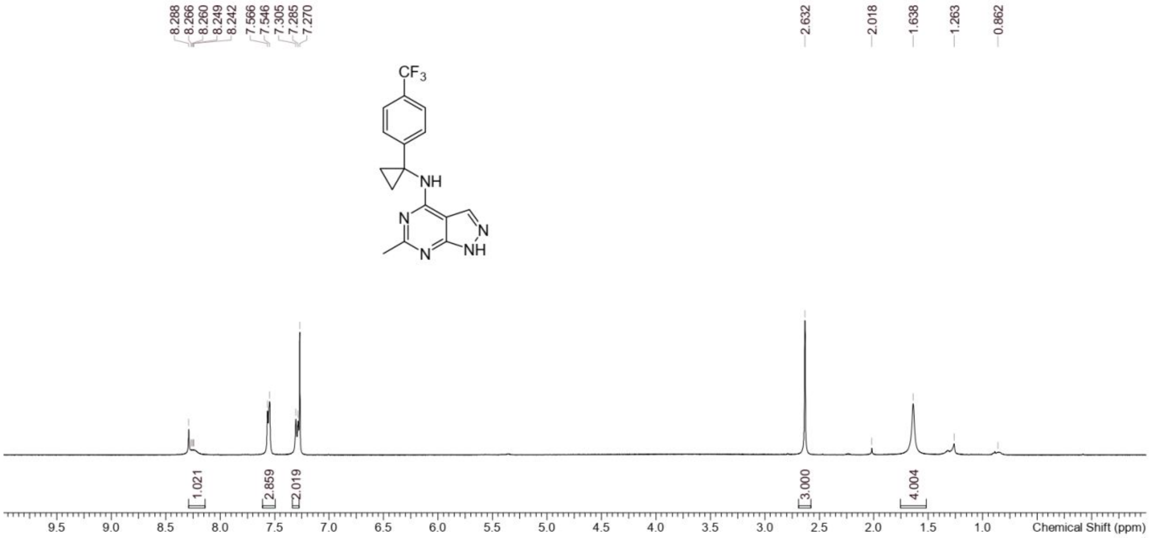

HPLC analysis of compound **1**

HPLC REPORT

Print time : 12/19/2023 14:24:01

Compound ID :1
Sample ID : EB13215-27-P1H1
Injection Date :2023/12/19 14:15:51
Injection Vol : 4ul
Location :tray1 vail8
Acq Method :0-60AB_8min_220&254&215.lcm
Org Data File :D:\DATA\2023\2312\231219\EB13215-27-P1H1.lcd
Instrument :HPLC_01 1-2402

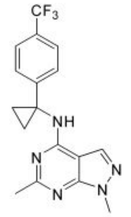

Chemical Formula: $C_{17}H_{16}F_3N_5$
Exact Mass: 347.14
Molecular Weight: 347.35

Chromatogram

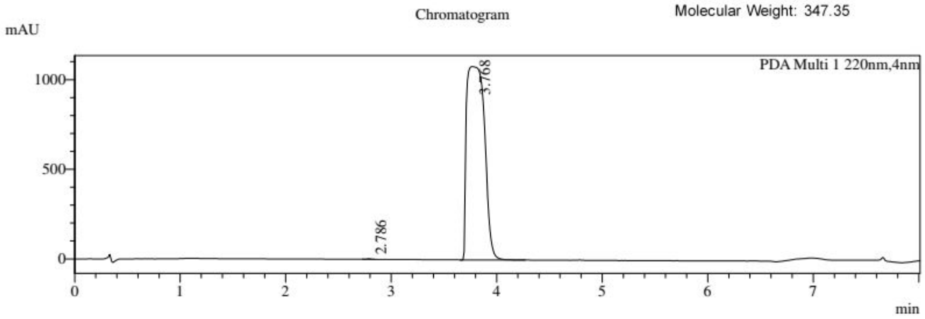

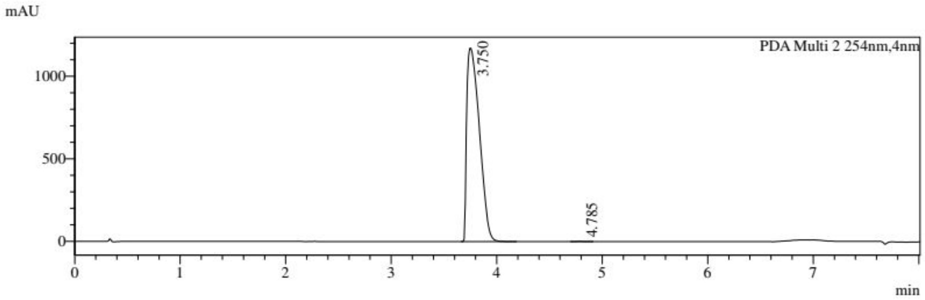

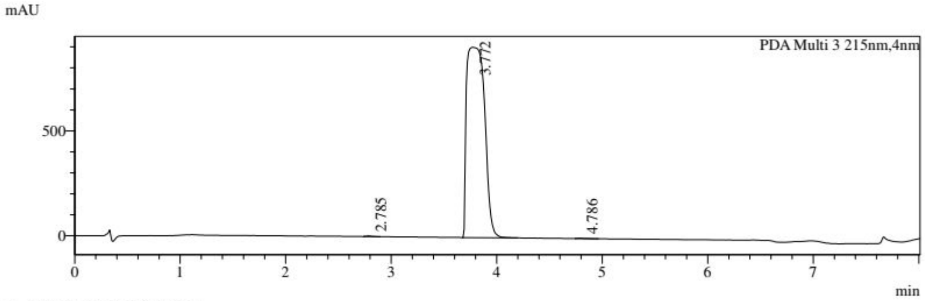

1 PDA Multi 1 / 220nm,4nm
2 PDA Multi 2 / 254nm,4nm
3 PDA Multi 3 / 215nm,4nm

Integration Result

Peak Table

PDA Ch1 220nm

Peak#	Ret. Time	Height	Height%	USP Width	Area	Area%
1	2.786	3010	0.278	0.087	8205	0.064
2	3.768	1078323	99.722	0.280	12808691	99.936

PDA Ch2 254nm

Peak#	Ret. Time	Height	Height%	USP Width	Area	Area%
1	3.750	1170589	99.822	0.232	9792746	99.933
2	4.785	2083	0.178	0.086	6552	0.067

PDA Ch3 215nm

Peak#	Ret. Time	Height	Height%	USP Width	Area	Area%
1	2.785	3284	0.360	0.089	8765	0.081
2	3.772	906601	99.443	0.268	10806868	99.860
3	4.786	1790	0.196	0.078	6384	0.059

MS analysis of compound **1**

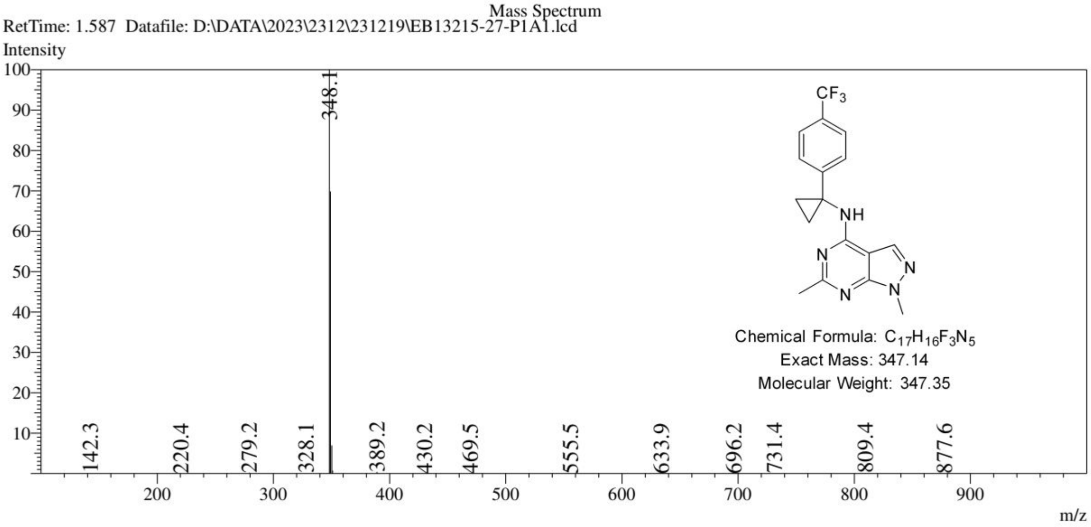

HRMS analysis of compound **1**

Sample Spectra

+ Scan (rt: 1.684-2.104 min) Sub

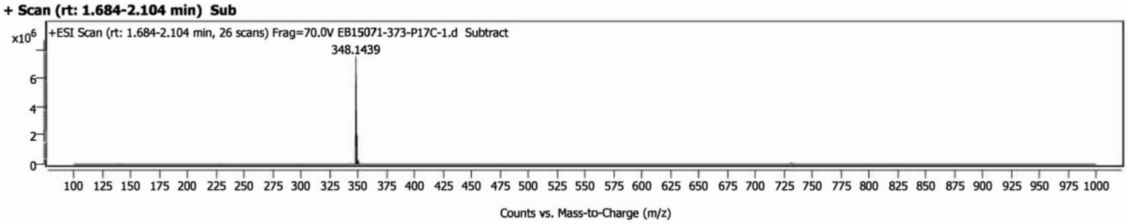

Spectrum Peaks

m/z	Z	Abund	Abund %	m/z (Calc)	Diff (ppm)	Ion Species	Formula	Ion Type
348.1439	1	7570354	100.00	348.1431	2.37	(M+H)+	C17 H16 F3 N5	
349.1466	1	2059542	27.21	349.1459	2.17	(M+H)+	C17 H16 F3 N5	
350.1494	1	202231	2.67	350.1487	2.16	(M+H)+	C17 H16 F3 N5	
348.2365		198101	2.62					

Spectrum Identification Table

Best ID Source	Name	Formula	Species	m/z	Diff (ppm)	CAS	Score	Score (Lib)	Score (DB)	Score (MFG)	Lib/DB
No MFG		C17 H16 F3 N5	(M+H)+	348.1439	2.33		90.00			90.00	

HPLC analysis of compound **9**

HPLC REPORT

Print time : 01/17/2024 13:42:10

Compound ID :9
Sample ID : EB13326-38-P1H1
Injection Date :2024/1/17 13:34:01
Injection Vol : 10ul
Location :tray1 vail16
Acq Method :10-80AB_8min_220&254&215.lcm
Org Data File :D:\DATA\2024\2401\240117\EB13326-38-P1H1.lcd
Instrument :HPLC_01 1-2402

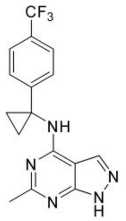

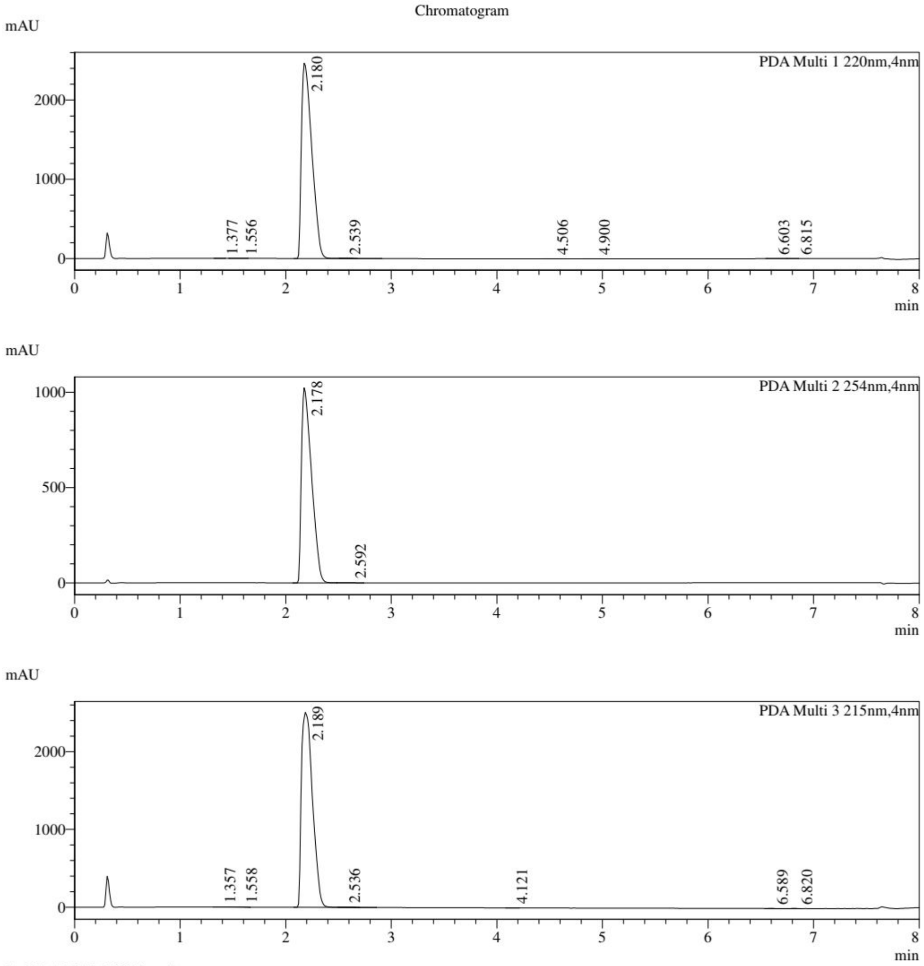

1 PDA Multi 1 / 220nm,4nm
2 PDA Multi 2 / 254nm,4nm
3 PDA Multi 3 / 215nm,4nm

==
Integration Result
==

Peak Table

PDA Ch1 220nm

Peak#	Ret. Time	Height	Height%	USP Width	Area	Area%
1	1.377	577	0.023	0.094	1865	0.011
2	1.556	1169	0.047	0.122	4275	0.025
3	2.180	2464289	99.784	0.203	16946604	99.860
4	2.539	741	0.030	0.169	4330	0.026
5	4.506	809	0.033	0.133	3815	0.022
6	4.900	770	0.031	0.149	3843	0.023
7	6.603	541	0.022	0.203	2962	0.017
8	6.815	736	0.030	0.096	2618	0.015

PDA Ch2 254nm

Peak#	Ret. Time	Height	Height%	USP Width	Area	Area%
1	2.178	1023013	99.929	0.202	6817782	99.889
2	2.592	731	0.071	0.397	7575	0.111

PDA Ch3 215nm

Peak#	Ret. Time	Height	Height%	USP Width	Area	Area%
1	1.357	628	0.025	0.123	2919	0.016
2	1.558	2083	0.083	0.104	6829	0.037
3	2.189	2505113	99.756	0.198	18360398	99.868
4	2.536	1236	0.049	0.208	8097	0.044
5	4.121	497	0.020	0.085	1405	0.008
6	6.589	807	0.032	0.088	1999	0.011
7	6.820	871	0.035	0.101	2944	0.016

MS analysis of compound **9**

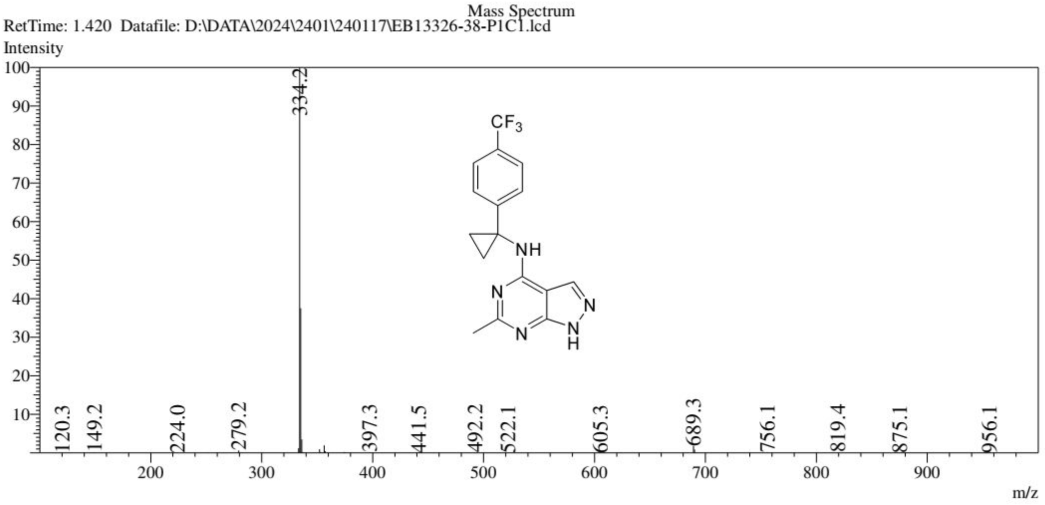

HRMS analysis of compound **1**

Sample Spectra

+ Scan (rt: 1.503-1.856 min) Sub   C16 H14 F3 N5

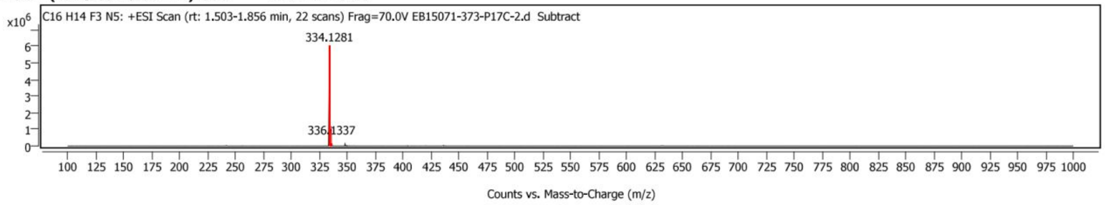

Spectrum Peaks

m/z	Z	Abund	Abund %	m/z (Calc)	Diff (ppm)	Ion Species	Formula	Ion Type
334.1281	1	6000415	100.00	334.1274	2.19	(M+H)+	C16 H14 F3 N5	
335.1309	1	1034757	17.24	335.1302	1.98	(M+H)+	C16 H14 F3 N5	
336.1337	1	97288	1.62	336.1329	2.28	(M+H)+	C16 H14 F3 N5	
348.1432	1	139281	2.32	348.1431	0.41			

Spectrum Identification Table

Best	ID Source	Name	Formula	Species	m/z	Diff (ppm)	CAS	Score	Score (Lib)	Score (DB)	Score (MFG)	Lib/DB
Yes	MFG		C16 H14 F3 N5	(M+H)+	334.1281	2.17		97.34			97.34	

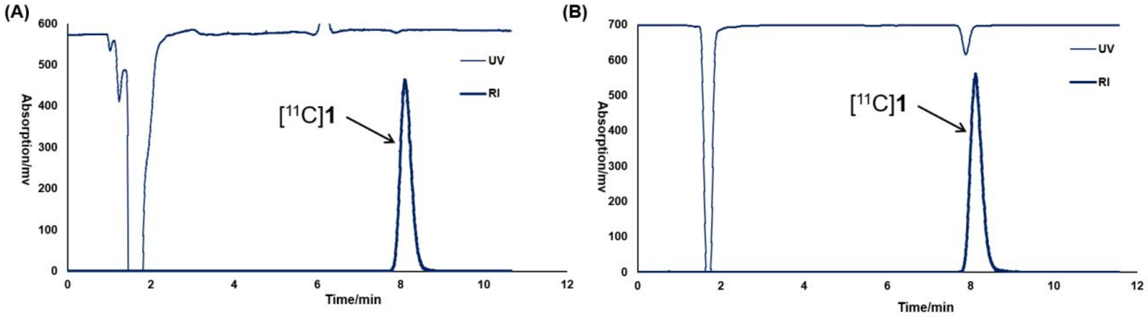

Figure S1. (A) HPLC chromatographic analysis of [^{11}C]**1**; (B) HPLC co-injection analysis of [^{11}C]**1** with the corresponding non-radioactive reference compound.
